# To evaluate and compare the effect of 17% EDTA, 10% citric acid, 7% maleic acid on the dentinal tubule penetration depth of bio ceramic root canal sealer using confocal laser scanning microscopy: an *in vitro* study

**DOI:** 10.12688/f1000research.127091.2

**Published:** 2023-02-24

**Authors:** Shivangi Shekhar, P. Laxmish Mallya, Vasudev Ballal, Ramya Shenoy

**Affiliations:** 1 Department of Conservative Dentistry and Endodontics, Manipal College of Dental Sciences, Manipal Academy of Higher Education, Manipal, Karnataka, 575001, India; 2 Conservative Dentistry and Endodontics, Manipal College of Dental Sciences, Mangalore, Manipal Academy of Higher Education, Manipal, Karnataka, 576104, India; 3 Department of Public Health Dentistry, Manipal College of Dental Sciences, Mangalore, Manipal Academy of Higher Education, Manipal, Karnataka, 575001, India

**Keywords:** Biofilms, Citric Acid, EDTA, Maleic Acid, Smear Layer.

## Abstract

**Background:** The main factors that affect the success of an endodontic infection are effective cleaning and shaping of the root canal system including complete disinfection by using chemical irrigating solutions and obturation with an endodontic sealer to provide a fluid-tight seal. Using rotary and hand instruments for shaping and cleaning the root canal along with irrigants produces a smear layer on the surface of the root dentin affecting the penetration of the endodontic sealer into the dentinal tubules. This smear is difficult to remove with the use of only endodontic irrigants, hence, chelating agents were introduced in adjunct with irrigating solutions for irrigation protocol for effective removal of smear layer which affect the penetration of endodontic sealers into the dentinal tubules.

**Methods:** 32 mandibular premolar teeth were used. The biomechanical preparation was done till Protaper F3 size. Irrigation was done with 2.5 mL sodium hypochlorite (NaOCl) solution after each instrumentation change for 1 min. Samples were then divided into 4 groups according to the irrigating solution used as the final rinse used with passive ultrasonic agitation. The groups were: Group I: 5 ml of saline, Group II: 5ml of 17% ethylenediaminetetraacetic acid, Group III: 5 ml of 10% citric acid, Group IV: 5 ml of 7% maleic acid each for one minute. All the canals were obturated with BioRoot ^tm^RCS with gutta-percha using the ultrasonic condensation technique. For staining the samples for Confocal LASER microscopy, BioRoot ^tm^RCS was mixed with Rhodamine B dye.

**Results:** The maximum penetration of bio-ceramic sealer was observed in the coronal region. At the apical third, the maximum sealer penetration was seen with 7% maleic acid.

**Conclusions:** Maximum sealer penetration was seen in the coronal section followed by the middle and apical section. Maximum sealer penetration was seen with 7 % maleic acid at the apical third.

## Introduction

The main purpose of an endodontic treatment is to eradicate the microorganisms, debris and necrotic tissue prevailing in the root canals and to provide effective cleaning and decontamination of the root canal system to avoid its re-contamination. ^1^ Chemomechanical preparation plays a significant role in the endodontic treatment. ^2^ The root canal at the apical region has a complex structure containing cul de sacs, lateral and accessary canals which makes the debridement of infected tissue from this area a challenging task. ^3^ Even after chemomechanical preparation using hand or rotary instruments, an average amount of 30-35% root canal area is left untouched and un-instrumented. Irrigating solutions do not reach this area and hence no proper cleaning of such areas leads to re-contamination and failure of the treatment. ^4^ The process of root canal cleaning and its shaping is established by biomechanical preparation and extensive irrigation using endodontic irrigants. ^5^ Chemo mechanical preparation using hand or rotary instruments produces a smear layer which is an uneven amorphous granular layer present on the root dentin containing both inorganic and organic contents like necrotic debris, odontoblastic processes, pulp tissue and microorganisms with their metabolic products. Although there is debate if the smear layer should be removed or kept, a recent meta-analysis and systematic review concluded that removing the smear layer improved the root canal system's fluid tight seal. ^6^ The smear layer has been demonstrated to be infected and protects the microorganisms present in the dentinal tubule and also inhibits the penetration of endodontic sealers and intracanal medicaments into the dentinal tubules which leads to some bacteria being left behind in the dentinal tubules which might later cause re-infection. ^7^

Irrigating solutions for endodontic treatment play a significant part in the success of the treatment. The process of instrumentation along with administration of the root canal irrigants, facilitates the elimination of microorganisms, smear layer, tissue fragments and necrotic debris present in the root canal completely by the flushing mechanism of action of the irrigants. ^8^ These irrigants have a benefit of reaching the accessory features and complex area such as lateral and accessory canals, and cul-de-sacs which are generally hard to access. Agitation and activation of the irrigants through lasers, sonics and ultrasonics are additional sources which help to improve the smear layer removal along with irrigants used for canal irrigation. ^9^ Passive ultrasonic irrigation (PUI) employs ultrasonic wave energy that is transmitted from a tip or file to the irrigant and produces cleaner canals. ^10^ To eradicate the smear layer, endodontists have used various chelating chemicals such as ethylenediamineactetic acid (EDTA), phosphoric acid, maleic acid (MA), citric acid (CA) and etidronic acid. The use of demineralizing agents along with sodium hypochlorite irrigation has been suggested for active removal of smear layer for successful endodontic treatment. For many years, 17% EDTA has been used as a gold standard chelating agent. The consequence of EDTA on dentin totally depends on the total time it is in contact with dentin and also on the concentration of EDTA solution. Several previous studies resolved that irrigation in the endodontic treatment with EDTA appears to be a promising endodontic tool. But in our study 17% EDTA was less effective when compared with MA and CA to eliminate smear layer successfully. The reason could be the higher surface tension of 17% EDTA. A previous study concluded that surface tension of 7% MA is lower compared to 17% EDTA. ^11^ Since EDTA is extremely active at a neutral pH and a decrease in pH over time is seen. Hence, its efficacy with time decreases as decrease in pH causes decrease in its effectiveness. Ballal *et al.* ^12^ in their research also concluded that that final irrigation during biomechanical preparation with 7% MA for one min was more active in eradicating the smear layer when compared to 17% EDTA at the apical region of the root canal. Demirel *et al.* in their study revealed that irrigation with 6 % citric acid was more efficient compared to 10% EDTA for eradicating smear layer present in root canal dentin. ^13^

A study done Mark et al aimed to assess the influence of 1% phytic acid and 17% ethylenediaminetetraacetic acid (EDTA) on the dentinal tubules penetration of EndoSequence BC bioceramic-based and AH Plus resin-based sealers using confocal laser scanning microscopy (CLSM). The study concluded that 17% EDTA solution has better penetration into dentinal tubules compared to 1% phytic acid. ^14^

S lottani et al aimed to evaluate the effects of ethylenediaminetetraacetic (EDTA), etidronic (EA) and peracetic acid (PA) when used in conjunction with sodium hypochlorite (NaOCl) as root canal irrigants on calcium eluted from canals, smear layer, and root dentine demineralization after instrumentation/irrigation and concluded that the decalcifying agents under investigation were all able to remove or prevent a smear layer. However, they eroded the dentine wall differently. ^15^

Since complete elimination of microorganisms from the endodontic space is unlikely, the antimicrobial activity of root canal sealers may help to remove residual microorganisms which remain unaffected by chemo mechanical preparation and irrigating solutions used for the root canal treatment. To date, no comparative studies have been published that compare the smear layer removal and the penetration depth of Bio-ceramic sealer into the dentinal tubules present in the root canal.

The goal of this *in vitro* study is to use a Confocal LASER scanning microscope to compare the effect of saline, EDTA, MA, and CA solutions on removal of smear layer and to measure depth of penetration of Bio ceramic sealer (BioRoot ^tm^RCS) into the dentinal tubules at the coronal, middle and apical third regions of the root canal. The null hypothesis tested is that there is no significant difference in smear layer and sealer penetration among saline, 7% MA, 10% CA and 17% EDTA when used as final irrigating solution.

## Methods

### Ethics

The ethical committee clearance was attained from the institutional (Manipal College of Dental Sciences) ethical committee (reference number-19083 (18/09/2019)).

### Sample

Sample size was based on Mead’s resource equation, ^16^ where the total number of samples in the study was based on blocking component, treatment component and error component. A total of 32 samples and per group 8 samples were included. 32 mandibular premolar teeth extracted for orthodontic purpose with straight roots, single canal and no resorption or caries were used. As these are routinely extracted teeth, they were easily obtained from the orthodontic clinic. Hence patient consent was not required.

The teeth used were kept in a solution of 0.2% sodium azide (Sigma Chemical Co, St Louis, MO) at 4°C. The occurrence of one canal in the teeth was confirmed radiographically on three films angulated at different angles. The teeth were decoronated at cementoenamel junction (CEJ) with a 0.1mm diamond disc to standardize the length of the root to 15 mm from the anatomic apex. ^16^ The working length (WL) of each tooth was calculated by introducing a 15 K file (Mani Inc, Tochigi Ken, Japan) into each canal until it was seen at the root apex and subtracting 1 mm from this point and then later confirmed with radiographs. Sizes 2 and 3 of Gates Glidden drill (Mani Inc, Tochigi Ken, Japan) were used to enlarge the coronal region for easier access to the middle and apical regions of the root canal. The apical region of each canal was enlarged till ISO 40 K file (Mani Inc, Tochigi Ken, Japan). The chemo mechanical preparation was done using ProTaper files till size F3 (Dentsply Protaper Universal) by applying the Crown Down technique and the master apical file chosen was F3. ^16^

Irrigation of the root canal was performed with 2.5 mL NaOCl (Vishal Dentocare Pvt. Ltd, INDIA) solution after each instrumentation change for one minute. ^17^

Samples were further divided into four groups with eight samples according to final irrigating solution used with passive ultrasonic agitation.

The groups were:

Group I: 5 ml of 0.9% physiological saline for 1 minute

Group II: 5ml of 17% EDTA for 1 minute

Group III: 5 ml of 10% CA for one minute for 1 minute

Group IV: 5 ml of 7% MA for 1 minute.

Irrigation was done using 2-ml disposable plastic syringe with a side vented 27-gauge needle (Dispovan, Mumbai, Maharashtra, India) which was inserted 1 mm short of the WL. Passive ultrasonic agitation was done after the irrigation with the irrigating solutions. For passive ultrasonic agitation, a side vented 30-guage was used. Ultrasonic file (Irrisafe, Satelec, Aceton Group, Merignac cedex, France) of size 20, 2% taper was introduced into the canal 1mm short of the apex. Ultrasonic activation was carried out for one minute with a power of 3 using an ultrasonic unit (Suprasson P5 Booster, Satelec). It was repeated three times to maintain a total span for three minutes. After the final irrigation with the solutions the root canals were dried up using paper points. BioRoot ^tm^ RCS, a bio-ceramic sealer (Septodont) was used for obturation. The sealer was applied to the canal walls using a lentino spiral and obturation using gutta percha was done using the lateral condensation technique. ^17^

For fluorescence under confocal microscopy, Rhodamine B dye (Loba Chemie Pvt. Ltd.) was mixed with BioRoot ^tm^ RCS sealer. After obturation the teeth were stored in a humidifier with 100% humidity for twenty-four hours which allowed the sealer to set in the presence of moisture. After 24 hours, all the sample were removed from the humidifier and were segmented at distances of 2 mm, 5 mm and 8 mm from the root apex equivalent to apical, middle and coronal regions of each tooth. ^16^ All the specimens were sectioned using a diamond disc at 90° to the long axis of the tooth with a straight handpiece and micromotor unit (Confident Dental Equipments Ltd.).

The sections were kept flat at 2mm thickness each for ease of mounting onto the glass slides for Confocal Scanning Electron Microscopy (CSLM). The specimens were kept on the glass slides provided and were then examined under confocal LASER scanning microscope. The confocal LASER microscope used was DPSS model DMi8. All the sections of the root specimen were scanned at 10×. The excitation was kept at 561 nm to collect the emission produced at 585-682 nm mode. Images obtained on the computer were then processed for background noise reduction (Leica Application Suite X, version 3.5.7.23225). Depth of penetration of sealer was recorded for each part and mean value of each section was calculated. Digital ruler inbuilt in the software (LAS-AF, Leica) was used for measuring the depth of sealer penetration. ^18^

### Data analysis

Statistical product and service solutions, version 20.0 (SPSS Inc., Chicago, IL, USA) was used in analysis. Statistical analysis was performed by using the one-way ANOVA. The *post hoc* Tamhanes test was applied for comparisons between the groups (intergroup analysis) and within the groups (intragroup analysis). The level of significance (P) was kept at 5%.

## Results

The result of the present study concluded that the maximum depth of Bio-ceramic sealer penetration of all the groups were seen at the coronal third followed by middle and apical third of the root specimens examined ( Table 1).

**Table 1.** Maximum and minimum depth of sealer penetration of irrigating solution.

Groups	Coronal 1/3 ^rd^	Middle 1/3 ^rd^	Apical 1/3 ^rd^
**Saline**	Min:392.42 Max: 663.51 Mean/SD: 520.02±83.84	Min: 208.52 Max: 459.15 Mean/SD: 316±62.19	Min:106.04 Max: 220.42 Mean/SD:154.80±30.38
**17% EDTA**	Min: 823.33 Max: 1260.26 Mean/SD: 1029.94±130.51	Min: 361.50 Max: 864.08 Mean/SD: 541.89±108.04	Min: 192.18 Max: 369.26 Mean/SD: 267.30±57.57
**10% Citric acid**	Min: 841.72 Max: 1174.31 Mean/SD: 976.61±96.36	Min: 546.01 Max: 995.70 Mean/SD: 747.28±129.71	Min: 258.14 Max: 647.71 Mean/SD:412.13±96.31
**7% Maleic acid**	Min: 902.37 Max: 1369.05 Mean/SD: 1077.95±124.74	Min: 527.50 Max: 998.70 Mean/SD: 769.37±126.39	Min: 241.90 Max: 563.37 Mean/SD: 348.11±63.88

The one way ANOVA shows a statistically significant difference between the groups and within the groups. Results showed statistical significance with the df 2; F value 2, P value- .000 ( Table 2). The results showed the saline group had a significantly lower depth of sealer penetration compared to the other groups in all the three sections of the root specimen (P < .05).

**Table 2.** One way ANOVA test for intragroup analysis at coronal, middle and apical third.

Irrigating solution	Values	Sum of squares	Df	Mean square	F	Sig
**SALINE**	Between groups	1607427.450	2	803713.725	203.965	.000
	Within groups	271890.804	69	3940.446		
	Total	1879318.254	71			
**17% EDTA**	Between groups	7673981.752	2	3836990.876	365.496	.000
	Within groups	724365.464	69	10498.050		
	Total	8398347.216	71			
**10% CITRIC ACID**	Between groups	3906708.467	2	1953354.234	165.595	.000
	Within groups	813922.001	69	11795.971		
	Total	4720630.468	71			
**7% MALEIC ACID**	Between groups	6442656.784	2	3221328.392	271.319	.000
	Within groups	819226.065	69	11872.842		
	Total	7261882.850	71			

The results of the inter group analysis demonstrated that there was a significant statical difference seen in the depth of penetration of sealer between all irrigating solutions at the coronal, middle and apical third of the root specimen (P < .05). No statistically significant difference in the depth of bio- ceramic sealer penetration between EDTA, CA, MA at coronal third was observed. At the middle and apical regions, a statistically significant difference was seen in the depth of penetration of sealer between EDTA and CA and MA ( Table 3).

**Table 3.** *Post hoc* Tamhanes test for intergroup analysis at coronal, middle and apical third.

	Group (I)	(J) Group	Mean difference (I-J)	STD. Error	SIG. (p<.05)
**CORONAL THIRD**	SALINE	EDTA	509.9350000	31.6791040	.000
		CITRIC ACID	50.3291667	33.1299042	.584
		MALEIC ACID	-48.0037500	36.8655085	.737
	EDTA	CITRIC ACID	-459.6058333	26.0730230	.000
		MALEIC ACID	-557.9387500	30.6802492	.000
	CITRIC ACID	MALEIC ACID	-98.3329167	32.1761187	.023
**MIDDLE THIRD**	EDTA	SALINE	225.3304167	25.4480542	.000
		CITRIC ACID	-22.185000	34.6915161	.000
		MALEIC ACID	-227.4775000	33.9418523	.000
	SALINE	CITRIC AC ID	-446.5154167	29.6358751	.000
		MALEIC ACID	-452.8079167	28.7547064	.000
	CITRIC ACID	MALEIC ACID	-6.2925000	37.1850906	1.000
**APICAL THIRD**	EDTA	SALINE	82.4970833	12.3952808	.000
		CITRIC ACID	-174.8300000	22.3990134	.000
		MALEIC ACID	-110.8112500	16.8892395	.000
	SALINE	CITRIC ACID	-257.3270833	20.6160024	.000
		MALEIC ACID	-193.3083333	14.4412661	.000
	CITRIC ACID	MALEIC ACID	64.0187500	23.5928587	.057

For intra group analysis, the *post-hoc* Tamhanes test results showed that there was a significant difference (P < .05) among all final irrigating solutions at different sections of the root specimen ( Table 4).

**Table 4.** *Post hoc* Tamhanes test for intragroup analysis at the coronal, middle, apical third.

Irrigating solutions			Mean difference (I-J)	Std. Error	Sig. (p<.05)
**SALINE**	CORONAL	MIDDLE	203.4475000	21.3092101	.000
		APICAL	365.2025000	18.2033561	.000
	MIDDLE	APICAL	161.755000	14.1303575	.000
**17% EDTA**	CORONAL	MIDDLE	488.0520833	34.5989402	.000
		APICAL	792.6404167	28.7375442	.000
	MIDDLE	APICAL	304.5883333	24.5271165	.000
**10% CITRIC ACID**	CORONAL	MIDDLE	232.3300000	32.9840900	.000
		APICAL	567.4812500	27.8111472	.000
	MIDDLE	APICAL	335.1512500	32.9785181	.000
**7% MALEIC ACID**	CORONAL	MIDDLE	308.5783333	36.2495296	.000
		APICAL	729.8329167	28.6088393	.000
	MIDDLE	APICAL	421.2545833	28.9087582	.000

For EDTA, the maximum sealer penetration depth was seen at coronal third which had no statistically significant difference with CA and MA, suggesting that all the three irrigating solutions were similarly effective in removal of smear layer at coronal third.

At the middle and apical third, a statistically significant difference was seen between EDTA and CA and MA suggesting that CA and MA were more active than EDTA in removal of smear layer at middle third, and hence greater penetration of Bio-ceramic sealer into the dentinal tubules were seen with CA and MA.

No statistical difference was seen between CA and MA at middle third and apical third suggesting that both were similarly effective in removal of smear layer hence influenced the penetration of bio ceramic sealer in the dentinal tubules.

**Figure 1.**
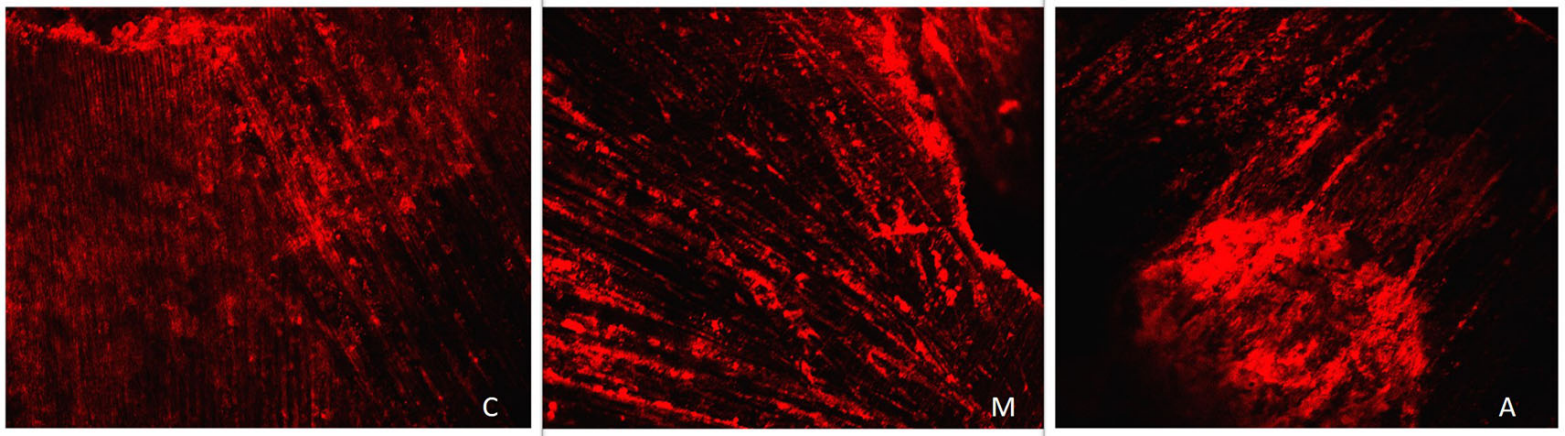
Representation of Confocal LASER scanning microscopic images from the Citric acid group. (C: Coronal, M: Middle, A: Apical).

**Figure 2.**
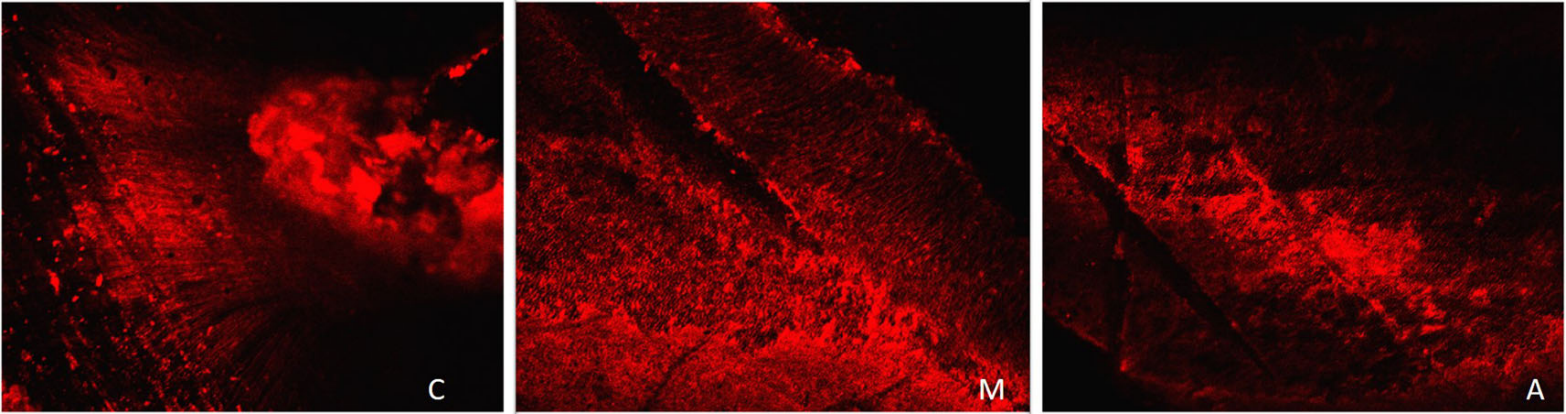
Representation of Confocal LASER scanning microscopic images from the saline group. (C: coronal, M: Middle, A: Apical).

**Figure 3.**
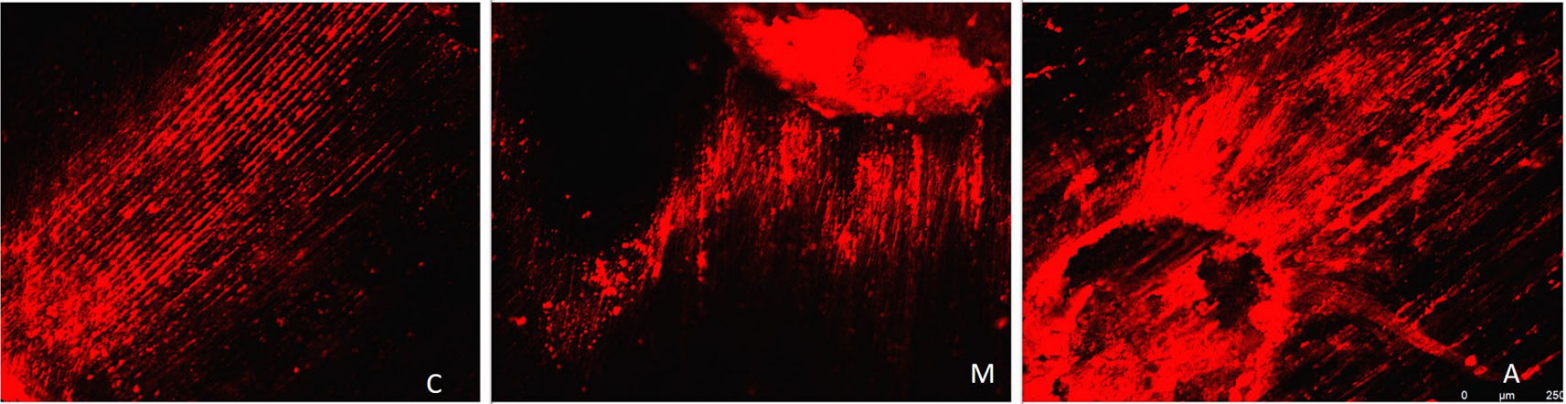
Representation of Confocal LASER scanning microscopic images from the Maleic acid group. (C: coronal, M: Middle, A: Apical).

**Figure 4.**
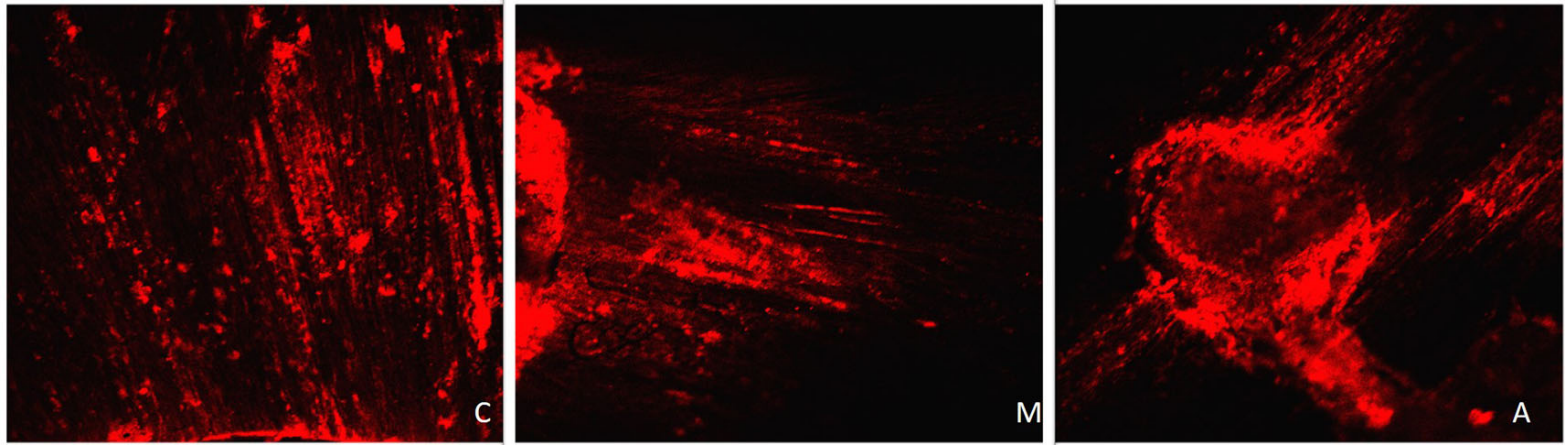
Representation of Confocal LASER scanning microscopic images from the Eythenediamine tetracetic acid group. (C: coronal, M: Middle, A: Apical).

Figure 1 displays a representation of Confocal LASER scanning microscopic images from the Citric acid group (C: Coronal, M: Middle, A: Apical). Figure 2 displays a representation of Confocal LASER scanning microscopic images from the Saline group. Figure 3 displays a representation of Confocal LASER scanning microscopic images from the Maleic acid group, and Figure 4 displays a representation of Confocal LASER scanning microscopic images from the Eythenediamine tetracetic acid group.

## Discussion

The current study was done to evaluate the efficacy of 17 % EDTA, 10% CA, 7% MA and 0.9% physiological saline in the depth of penetration of bio ceramic sealer into the dentinal tubules of different sections of the root specimens. To date, not much evidence has been published for penetration depth of Bio-ceramic sealer into the dentinal tubules of root canal dentin. Hence, this study included a Bio-ceramic sealer as they set even in the presence of moisture not affecting its properties.

The literature shows that most irrigants used for irrigation along with biomechanical preparation are not effectively active against smear layer at all the sections of the root specimen, particularly at the apical region, which is vital in determining the prognosis of an endodontic treatment. The results of this study revealed that both 7% MA and 10% CA were equally effective in removing smear layer at middle and apical third. To facilitate a satisfactory cleaning and infiltration of irrigants into the dentinal tubules, the apical region of each specimen was enlarged until size 40 no K file. This was done in agreement with other studies that have concluded that a greater apical preparation allows a greater decrease in residual bacteria and aids in better smear layer removal as compared with smaller preparation. ^19^ Passive ultrasonic agitation was done to produce acoustic streaming to eliminate debris from the canal space and leave behind cleaner canals. Passive ultrasonic agitation when used in combination with conventional syringe irrigation produces clear canals. This is in agreement with earlier studies. ^11^ The most important factors that affect the action of the irrigating solution are the concentration of the irrigant used and its contact time with the root canal dentin. The exact optimum contact time needed for the irrigant to be held in reserve in root canals for effective removal of smear layer is unclear. Studies have shown that EDTA reasonably removes the smear layer in one min, but it also produced extreme erosion of intertubular and peritubular dentin when it was applied for duration of more than 10 mins. ^20^ Ballal *et al.* ^12^ in their study concluded that final irrigation for one min with 7% MA was effective at the apical third in smear layer removal. Therefore, the irrigation time of one min was kept for the irrigants used in this particular study.

The results of the present study revealed that 7% MA and 10% CA had greater depth of bio ceramic sealer penetration into the dentinal tubules suggesting they had a better capability for removing smear layer when compared to 17% EDTA in the apical and middle third regions of the root specimen. No statistically significant difference was seen in the depth of the Bio-ceramic sealer penetration between 7% MA and 10% CA at the middle and apical third suggesting that at apical third both the irrigants were equally effective in eliminating the smear layer. This result is in accordance with other studies. ^21^^,^^22^

EDTA is a chelating solution which aids in the elimination of smear layer by facilitating the elimination of microorganisms present in the canal space, thereby improving the anti-microbial effect of disinfecting agents in further deeper layers of dentin. The consequence of EDTA on dentin totally depends on the total time it is in contact with dentin and also on the concentration of EDTA solution. Several previous studies resolved that irrigation in the endodontic treatment with EDTA appears to be a promising endodontic tool. ^23^ However, in our present study, the depth of sealer penetration into dentinal tubules with EDTA was lower at the middle and the apical third when compared to 7% MA and 10% CA suggesting it was less effective in removing the smear layer at these areas. Even after larger apical preparation, EDTA was less effective when compared with MA and CA to eliminate smear layer successfully. The reason could be the higher surface tension of 17% EDTA. A previous study concluded that surface tension of 7% MA is lower compared to 17% EDTA. ^11^ Since EDTA is extremely active at a neutral pH and a decrease in pH over time is seen. Hence, its efficacy with time decreases as decrease in pH causes decrease in its effectiveness. ^11^ Goldberg *et al.* concluded that optimal results are obtained only after an application time of minimum 15 mins with EDTA, but in this study the application time was only one minute hence EDTA was not as effective removing smear at the apical third. ^24^ Even though some studies have reported better chelating agents than 17% EDTA, contraindicatory studies still state that 17% EDTA is as effective as 10% citric acid or its even better as chelating agent. ^25^

Research done by Banode *et al.* ^26^ presented that the final irrigation with citric acid showed better results compared to EDTA in removing the smear layer from the canal space. In this present study, 10% citric acid solution was used because of its biocompatibility in addition to its ability to eliminate microorganisms, infected tissue and inorganic smear layer present in the root canal dentin. This is in accordance with study done by Malheiros ^27^ which concluded that 10–25% citric acid has better biocompatibility compared to 17% EDTA. The citric acid used was effective in all sections of the tooth. This result is in accordance with Schafer’s declaration that 1–40% citric acid can be used as an irrigant for endodontic treatment as citric acid is an organic acid which has the ability to demineralize the sclerosed dentin present at the apical region of the root canal. ^28^

MA produces a better demineralizing effect in a briefer period of time because it’s highly acidic at lower pH within a shorter period of time as it has a greater demineralizing effect when compared to EDTA. As stated above, the dentin present at the apical region of the root canal is greatly sclerosed. The surface tension of 7% maleic acid is less when compared to EDTA hence its acidity doesn’t increase, and it remains highly acidic at low ph. The maleic acid as stated above has the ability of eliminating the smear layer and demineralising inter and peri tubular dentin is because of its low pH of 1.05. Hence, the maleic acid used in this study was efficient in eliminating smear layer from all the sections of the root specimen. There was no statistically significant difference seen between 7% MA, 10% CA and 17% EDTA at the coronal third. The results showed no statistical significance between 7% MA and 10% CA at apical and middle third, concluding that both were better than 17% EDTA in eradicating smear layer at those sections of the root specimen. For the above reasons, better smear layer removal and depth of penetration of Bioceramic sealer was seen with MA and CA at the apical and middle sections significant with the other groups. All the specimens analysed for the control group were heavily smeared in all the sections (coronal, middle and apical third) of the root specimen. ^28^ Hence the null hypothesis was rejected.

The maximum penetration with Bio-ceramic sealer was seen in the coronal section of the root specimen for all the tested irrigants solutions concluding that better smear layer removal was seen at the coronal third. The result is in agreement with the previous study done by Kara *et al.* which determined that the supreme penetration depth of an endodontic sealer was seen in the coronal region when compared to the apical region and various other studies. ^29^ This may be due to larger diameter at the coronal and middle third areas, allowing an improved movement of the irrigating solutions. ^13^ Difficulty in cleaning the apical most portion of the root canal can be explained by decrease in the diameter of the root canal at the apex, which decreases the access of irrigants at the apex which consequently results in reduction of its flow. These features don’t allow the irrigants to reach the entire working length and hence the apical area remains uncleaned. Irfan *et al.* concluded that features that could have an impact in the cleaning of the apical area of the root canals are the complex anatomical configuration seen at the apex, limited space available, low permeability and difficulty of access. ^30^

A study done by Alim at al aimed to investigate the effects of different chelation solutions on the penetration of resin-based and bioceramic root canal sealers into dentinal tubules using a device that assembles conventional microplate detection with automated digital microscopy. The final irrigation solutions used were saline (control), ethylenediaminetetraacetic acid (EDTA), maleic acid (MA), and etidronic acid (HEBP). They concluded that maximum sealer penetration was seen at coronal third of the root specimen. ^31^

The Bio-ceramic sealer used for the study was BioRoot RCS. It is a mineral based permanent sealer used for obturating the root canal. It has an excellent adhesion to dentin and also to the gutta percha cones. It is hydrophilic in nature and continues its adhesion even in the presence of moisture. As it was an *in vitro* study, the exact *in vivo* conditions could not be simulated. Confocal LASER microscopy is a well-known light microscopical technique for imaging fluorescently dyed samples with significant three-dimensional structure. It has the ability to capture images at dissimilar depths in a model to enable to reform the three-dimensional structures within an object. Samples used are generally treated with fluorescent dyes to make objects visible. Confocal microscopy delivers the capacity for non-invasive, direct, serial optical sectioning of living specimens in lateral resolution. ^32^

A study done by Shaimaa et al aimed to evaluate the sealer penetration after applying dual rinse irrigant in comparison with sodium hypochlorite and 17% EDTA. Mandibular premolars were prepared by ProTaper Next and irrigated with NaOCl, NaOCl/Dual Rinse or NaOCl/EDTA and then obturated with a single-cone technique and bioceramic-based. Samples were observed using confocal laser microscopy, and the maximum depth of penetration was measured, as well as the percentage of sealer coating the canal wall and the penetration area of the sealer. The study concluded that Group (NaOCl/Dual Rinse) displayed better sealer penetration inside the dentinal tubules than Group (NaOCl/EDTA), while Group (NaOCl) showed the lowest sealer penetration. ^33^

## Conclusion

Maximum sealer penetration was seen in the coronal section followed by middle and apical sections of the root specimen. 17% EDTA, 10% CA, 7% MA were equally effective in sealer penetration at coronal third. However, in middle and apical section, 10% CA and 7% MA showed better efficacy in removal of smear layer compared to 17% EDTA, thereby showing more sealer penetration. Saline showed the least penetration of sealer in the dentinal tubules. The Bio-ceramic sealer used for the study was BioRoot RCS. It is a mineral based permanent sealer used for obturating the root canal and various have concluded that Bio-cermaic sealer sets in the presence of moisture and provides excellent adhesion to root dentin. A study done by Haji TH et al concluded that BioRoot sealers have excellent sealing adaptation and biocompatibility, as well as rapid tissue recovery.

## Data Availability

Figshare: Raw Data, https://doi.org/10.6084/m9.figshare.21280152.v7. ^34^ This project contains the following underlying data:
- RAW DATA OF SPECIMENS EXAMINED.xlsx (raw data as values of depth of sealer penetration into the dentinal tubules measured by an in-built ruler in mm in Confocal Laser Scanning Microscopy) - JPEG images of coronal, middle and apical third sections of the root specimen. Data are available under the terms of the Creative Commons Attribution 4.0 International license (CC-BY 4.0).
