# Supplementary material for: To evaluate and compare the effect of 17% EDTA, 10% citric acid, 7% maleic acid on the dentinal tubule penetration depth of bio ceramic root canal sealer using confocal laser scanning microscopy: an in vitro study
Source: F1000Res. 2023 Feb 24;11:1561. Originally published 2022 Dec 22. [Version 2] doi: 10.12688/f1000research.127091.2 (PMC9978241; doi:10.12688/f1000research.127091.2)
Supplement: Peer review history

# Peer review: Reviewer response for version 2

**DOI:** 10.5256/f1000research.144757.r164539

**Published:** 2023-03-01

**Authors:** Amber Ather

**Affiliations:** 1 Department of Endodontics, Virginia Commonwealth University School of Dentistry, Richmond, VA, USA

All the suggestions have been taken care of.

Is the work clearly and accurately presented and does it cite the current literature?

Partly

If applicable, is the statistical analysis and its interpretation appropriate?

I cannot comment. A qualified statistician is required.

Are all the source data underlying the results available to ensure full reproducibility?

Yes

Is the study design appropriate and is the work technically sound?

Yes

Are the conclusions drawn adequately supported by the results?

Partly

Are sufficient details of methods and analysis provided to allow replication by others?

Yes

Reviewer Expertise:

Endodontics

I confirm that I have read this submission and believe that I have an appropriate level of expertise to confirm that it is of an acceptable scientific standard.

# Peer review: Reviewer response for version 2

**DOI:** 10.5256/f1000research.144757.r164538

**Published:** 2023-03-01

**Authors:** Saravana Karthikeyan Balasubramanian

**Affiliations:** 1 Department of Conservative Dentistry and Endodontics, SRM Dental College, Ramapuram, SRM Institute of Science and Technology, Chennai, Tamil Nadu, India

The authors have done the suggested amendments in this revised version and hence, the manuscript can be accepted in its current form,

Is the work clearly and accurately presented and does it cite the current literature?

Yes

If applicable, is the statistical analysis and its interpretation appropriate?

I cannot comment. A qualified statistician is required.

Are all the source data underlying the results available to ensure full reproducibility?

Yes

Is the study design appropriate and is the work technically sound?

Yes

Are the conclusions drawn adequately supported by the results?

Yes

Are sufficient details of methods and analysis provided to allow replication by others?

Yes

Reviewer Expertise:

Dentistry, Endodontics, Tissue Engineering

I confirm that I have read this submission and believe that I have an appropriate level of expertise to confirm that it is of an acceptable scientific standard.

# Peer review: Reviewer response for version 1

**DOI:** 10.5256/f1000research.139564.r160806

**Published:** 2023-02-13

**Authors:** Amber Ather

**Affiliations:** 1 Department of Endodontics, Virginia Commonwealth University School of Dentistry, Richmond, VA, USA

Thanks for giving me an opportunity to review this paper by Shekhar *et al.* reporting on an important topic in the field of endodontics. This manuscript is focused on comparing different irrigant solutions and its effect on penetration depth of sealer.

 Please find my suggestions and concerns with manuscript below:

  

1. There are at least a few other papers published in literature comparing irrigants and its effect on the penetration of bioceramic sealer in human teeth. The literature search needs to redone and should include all the relevant data. Some of the relevant papers are as follows:
  - Alim Uysal BA, Kotan G, Guneser MB, Dincer AN, Senturk H, Rafiqi AM. Investigation of the effect of different chelation solutions on penetration of resin-based and bioceramic sealers with a novel method. *Microsc Res Tech*. 2021;84(7):1571-1576. doi:10.1002/jemt.23717
    Study has looked at various chelating solutions including Maleic acid on penetration of bioceramic sealer. Please include this study in the introduction and/or discussion section.
  - Eskander M, Genena S, Zaazou A, Moussa S. Effect of phytic acid and ethylenediaminetetraacetic acid on penetration depth of bioceramic and resin sealers. *Aust Endod J*. 2021;47(3):506-511. doi:10.1111/aej.12513
    This study is focused on the same so authors are requested to include this as well.  
  - Gawdat SI, Bedier MM. Influence of dual rinse irrigation on dentinal penetration of a bioceramic root canal sealer: A Conofocal microscopic Analysis. *Aust Endod J*. 2022;48(3):481-486. doi:10.1111/aej.12599
2. There are several errors with regard to references, which needs to be corrected.
  - E.g. authors mention reference #6 as a systematic review and meta-analysis in their manuscript; however, it’s an in vitro study. The authors are requested to refrain from using indirect references and cite the original paper. Similarly, in “Introduction” section, paragraph 3 ^rd^, line 3, authors have stated an author “Vibha et al” and have ended the sentence with reference #14. Reference #14 corresponds to “Loganathan M” in the bibliography. These are just few of the referencing and bibliography errors.
3. In “Materials and methods”, for root canal preparation, the authors mention the use of size 40 hand file to Working length and then use of Protaper F3 for final preparation. If apical prep was already done till size 40/02, then Protaper F3 corresponding to size (30/09 variable taper) will not alter the apical prep. The authors are requested to clarify the instrumentation sequence and the final Master apical file.
4. In Discussion section, the literature published on bioceramic sealer penetration needs to be discussed and compared with the study’s findings.
5. Please include the limitations of the study.
6. The conclusion does not include the focus on “bioceramic sealer”. Please rephrase the conclusion, as according to authors this is what makes their research novel.

Is the work clearly and accurately presented and does it cite the current literature?

Partly

If applicable, is the statistical analysis and its interpretation appropriate?

I cannot comment. A qualified statistician is required.

Are all the source data underlying the results available to ensure full reproducibility?

Yes

Is the study design appropriate and is the work technically sound?

Yes

Are the conclusions drawn adequately supported by the results?

Partly

Are sufficient details of methods and analysis provided to allow replication by others?

Yes

Reviewer Expertise:

Endodontics

I confirm that I have read this submission and believe that I have an appropriate level of expertise to confirm that it is of an acceptable scientific standard, however I have significant reservations, as outlined above.

## References

[1] : Investigation of the effect of different chelation solutions on penetration of resin-based and bioceramic sealers with a novel method. *Microsc Res Tech* .2021;84(7) : 10.1002/jemt.23717 1571-1576 10.1002/jemt.23717 33506518

[2] : Effect of phytic acid and ethylenediaminetetraacetic acid on penetration depth of bioceramic and resin sealers. *Aust Endod J* .2021;47(3) : 10.1111/aej.12513 506-511 10.1111/aej.12513 33811400

[3] : Influence of dual rinse irrigation on dentinal penetration of a bioceramic root canal sealer: A Conofocal microscopic Analysis. *Aust Endod J* .2022;48(3) : 10.1111/aej.12599 481-486 10.1111/aej.12599 34919319

# Response

**Published:** 2023-02-22

**Authors:** P. Laxmish Mallya

**Affiliations:** Manipal University, India

Noted Sir. Thank you for the corrections. I will try to upload a new version of the manuscript with all the corrections. Thank you Sir.

# Peer review: Reviewer response for version 1

**DOI:** 10.5256/f1000research.139564.r158693

**Published:** 2023-02-07

**Authors:** Saravana Karthikeyan Balasubramanian

**Affiliations:** 1 Department of Conservative Dentistry and Endodontics, SRM Dental College, Ramapuram, SRM Institute of Science and Technology, Chennai, Tamil Nadu, India

Thank you for rendering an opportunity to review the current article for the reputed journal, F1000Research. This interesting clinically significant study compared the effect of various root canal chelating agents by ultrasonic agitation on the dentinal tubule penetration of BioRoot ^tm^RCS , a bioceramic root canal sealer. However, this manuscript requires minor modifications before indexing. Please find my comments attached herewith:

 Abstract: 

- Background, Line 5: The authors need to mention as smear layer rather smear. Also, please state the aim/rationale of the study. 
- Methods: Please mention as 32 decoronated mandibular premolars.
- Results: The authors are suggested to briefly address the mean penetration values of the BioRoot RCS following the use of various irrigants at the coronal, middle and apical thirds. If not, please mention the mean values of significant groups atleast. The authors also need to highlight the type of statistical tests and the significance level employed in the study.
- Conclusions: The authors can suggest some recommendations as well about the use of maleic acid when compared to other irrigants. 

Manuscript:

- Introduction: Please highlight the drawbacks of 17% EDTA, the gold standard chelating agent. This further validates the rationale for the use of alternative chelating agents like CA and MA. 
- Discussion: Though, authors had well justified their results, it is recommended that the contraindicatory studies that favor the use of EDTA also need to be highlighted. 
- Acceptance/Rejection of Null hypothesis based on the current study results need to be done.
- Limitations of the study can be mentioned.
- The authors had mentioned that future in vitro studies are required pertaining to the use of 10% CA alone. They can also mention about the detailed exploration of the comparative studies between the three chelating agents as well.
- And the scope of the study that includes future ex vivo studies or in vivo animal studies can be addressed.  

Is the work clearly and accurately presented and does it cite the current literature?

Yes

If applicable, is the statistical analysis and its interpretation appropriate?

I cannot comment. A qualified statistician is required.

Are all the source data underlying the results available to ensure full reproducibility?

Yes

Is the study design appropriate and is the work technically sound?

Yes

Are the conclusions drawn adequately supported by the results?

Yes

Are sufficient details of methods and analysis provided to allow replication by others?

Yes

Reviewer Expertise:

Dentistry, Endodontics, Tissue Engineering

I confirm that I have read this submission and believe that I have an appropriate level of expertise to confirm that it is of an acceptable scientific standard, however I have significant reservations, as outlined above.
